# Autoinflammatory syndromes: diagnosis and management

**DOI:** 10.1186/1824-7288-36-57

**Published:** 2010-09-03

**Authors:** Sara De Sanctis, Manuela Nozzi, Marianna Del Torto, Alessandra Scardapane, Stefania Gaspari, Giuseppina de Michele, Luciana Breda, Francesco Chiarelli

**Affiliations:** 1 Department of Pediatrics, University of Chieti, Via Vestini 5, 66100 Chieti, Italy

## Abstract

During the last decades the description of autoinflammatory syndromes induced great interest among the scientific community. Mainly rheumatologists, immunologists and pediatricians are involved in the discovery of etiopathogenesis of these syndromes and in the recognition of affected patients. In this paper we will discuss the most important clues of monogenic and non-genetic inflammatory syndromes to help pediatricians in the diagnosis and treatment of these diseases.

## Introduction

The first time that a periodic disease was mentioned in the medical literature was in 1802 when Heberden described a condition characterized by recurrent abdominal pain, thoracic pain and painful extremities. Later several syndromes with recurrent episodes of fever and inflammatory symptoms were recognized, some of which with Mendelian inheritance pattern [1,2]. The term hereditary recurrent fever syndrome was associated to this group of syndromes; attacks begin early, usually before 10 years of age and recur with irregular intervals of time [3]. During the past decade, the term "autoinflammatory syndromes" was introduced by Kastner to include all those disorders that did not fit into classical groups of immune-mediated diseases, and characterized by recurrent fever associated with rheumatologic symptoms involving joints, skin, muscles, and eyes. The main difference with autoimmune diseases is that neither autoantigens nor autoantibodies are involved. Autoinflammatory syndromes are associated to a disregulation of the innate immune response with subsequent episodes of acute spontaneous inflammation [3]. This concept was initially assigned to the hereditary recurrent fevers but now is expanding to a broad number of disorders.

Thanks to the advanced techniques in genetics, to date many genes have been recognized in the pathogenesis of periodic syndromes like familial Mediterranean fever (FMF), hyper IgD syndrome (HIDS), tumor necrosis factor receptor associated autoinflammatory syndrome (TRAPS), cryopyrin associated periodic syndromes (CAPS), Blau syndrome and pyogenic sterile arthritis pyoderma gangrenosum and acne syndrome (PAPA). Some of these genes are still available for the molecular diagnosis, especially in patients with familial recurrence or with highly suggestive clues. Gattorno and collegues identified major clinical clues to predict the possibility of a genetic alteration in a suspected autoinflammatory syndrome. Early onset, family history of periodic fever, thoracic and abdominal pain, diarrhea and oral aphtous ulcers represent the most relevant clues to predict the possibility of a positive genetic test. The authors also proposed a flow chart based on the clinical profile of patients with suspected autoinflammatory syndromes with the purpose to identify patients with effective need of a genetic analysis. Thanks to this method, the number of genetic tests and relative costs have been significantly reduced [4].

To date few periodic syndromes remain of unknown origin like PFAPA syndrome (periodic fever, aphtous stomatitis, pharyngitis and adenitis), characterized by periodic fever attacks with early onset, of non-infective origin, with benign prognosis in the absence of familial inheritance [5] and Majeed syndrome, characterized by recurrent multifocal osteomyelitis (CRMO), congenital dyserithropoietic anemia, and neutrophilic dermatosis.

Epidemiological data about inflammatory diseases are poor; generally they are quite rare diseases. No gender prevalence has been described; only some reports recorded mild predominance in males for FMF [6]. The prevalence of other autoinflammatory syndromes is lower than FMF, but not yet quantified.

There are not specific and standardized therapies for the acute attacks of fever. On the other way long term treatment is available, effective in reducing the number and intensity of attacks and preventing severe complications like amyloidosis.

Acute attacks, especially in FMF, usually need a combination of drugs like non steroidal anti-inflammatory drugs (NSAIDs) and analgetics.

The proven effectiveness of anti IL-1 drugs in many autoinflammatory disorders made these drugs as first line choice in long-term treatment.

### Familial Mediterranean fever (FMF)

FMF, first described in 1945 by Siegal, is the most common autoinflammatory syndrome. It is an autosomal recessive inherited condition, prevalent among people of Mediterranean descent (Arabs, Turks, Armenians, non-Ashkenazi and Sephardic Jews) but may affect any ethnic group [7]; its prevalence is high among Sephardic Jews (100-400 every 100000 inhabitants) and quite low in west Europeans (2.5 per 100000) [3].

FMF is caused by mutations in the *MEFV *(MEditerranean FeVer) gene, located on the short arm of chromosome 16 (16p13.3). At least 100 disease-linked mutations in the MEFV gene have been described, the most being clustered in the 10th exon of this gene. The MEFV gene encodes for a protein of 781 aminoacids known as pyrin (or marenostrin). Pyrin is primarily expressed in neutrophils, eosinophils, monocytes, dendritic cells and fibroblasts. Wild-type pyrin suppresses inflammasome-mediated IL-1β production. Furthermore, wild-type pyrin plays an antiapoptotic role through inhibition of NF-kB nuclear trascription factor [8]. The most commonly reported mutations of MEFV gene are M694V, M680I and V726A. The presence of M694V mutation has been associated with more severe disease course and in particular with the development of amyloidosis [9].

FMF is clinically characterized by recurrent attacks of high spiking fever with associated serositis that usually last 1-3 days and subside spontaneously. Frequency of attacks is highly variable and asymptomatic periods lasting a few years have been reported [3]. Triggers may include stress, menstrual cycle, exercise and subclinical infections [7]. Recurrent fever may be the only manifestation in childhood [10]. Abdominal pain is present in 95% of cases and is associated with board-like rigidity of abdominal muscles, rebound tenderness, constipation or diarrhea [7,10]. Multiple air-fluid levels may be present on radiologic examination, leading to the suspicion of an acute abdomen [6]; the severity of the peritoneal episodes may mimic acute appendicitis and many patients have undergone appendicectomy [11]. Chest pain because of pleuric or pericardial involvement occurs in 33-53% of subjects. Patients with pleural involvement may have shallow breathing and dyspnoea. Musculo-skeletal involvement is frequent and characterized by myalgia, arthritis and/or arthralgia. Arthritis occurs in about 25 to 41% of cases [10]. It may be divided into three groups: 1) asymmetrical non destructive arthritis, in which one or two joints are swollen (most frequent by knees, ankles, and wrists) 2) chronic destructive arthritis, including sacroiliitis, 3) migratory polyarthritis.

Splenomegaly and scrotal pain may be present in pre-pubertal children during attacks. Thirty per cent of patients experience painful erysipela-like skin lesions usually on the distal extremities. A number of other different cutaneous manifestations such as Henoch-Schönlein purpura and polyarteritis nodosa can also occur during fever episodes [2,10,11].

Laboratory investigations during acute attacks are non-specific: leukocyte count, sedimentation rate and C-reactive protein (CRP) are often elevated during flares [2].

Amyloidosis is the most significant complication of FMF and may result in a progressive accumulation of serum amyloid A protein (SAA) mainly in the kidney. The most common clinical manifestation of amyloidosis is the development of proteinuria that may lead to renal failure. Patients are frequently normotensive and do not present hematuria. Before the advent of colchicine, the prevalence of amyloidosis among FMF patients was high ranging from 60% to 80% [12]. Renal involvement is usually observed after a variable time from disease onset. In few patients (less than 1%), renal amyloidosis may represent the first manifestation of the disease [6].

Acute attacks of FMF usually respond to a combination of non-steroidal anti-inflammatory drugs (NSAIDs) and analgesics. Colchicine is the treatment of choice for FMF. It greatly reduces the frequency and intensity of clinical attacks and prevents the development of renal amyloidosis. In adults the dose is 1 mg/day. In children the starting dose should be ≤0.5 mg/day for children below 5 years of age, 1 mg/day for children 5-10 years, and 1.5 mg/day for children above 10 years. Dosage can be increased in a stepwise fashion up to a maximum of 2 mg/day [2]. Only 5% of patients are non responders. Lack of response to colchicine is often caused by poor adherence to treatment [3].

Biologic treatments (anti TNF-α, anti IL-1 β) have been proposed in non responsive patients, although their efficacy has not been proved [2,10,13].

### Hyper IgD syndrome (HIDS)

Hyper IgD syndrome is an autosomal recessive disease characterized by recurrent fever attacks, preceded by chills and malaise; it was identified in 1984 in 6 patients with a history of recurrent fever and elevation of serum IgD. This syndrome seems to be more prevalent in Caucasians, especially in western Europeans [14]. Since it was first described, about 180 cases have been reported, and the majority of them living in France and Netherland [3,15].

HIDS is linked to mutations in the *MVK *gene (locus 12q24) which encodes the mevalonate kinase (MVK) [16], mainly expressed in peroxysomes. This enzyme is responsible of converting mevalonic acid into 5-phosphomevalonic acid which is a fundamental precursor of sterols (cholesterol, steroid hormones, vitamin D, bile salts) and isoprenoids. In HIDS patients residual enzyme activity of MVK is reduced to 1-8%, so the metabolic pathway of steroids synthesis induces lower levels of serum cholesterol, higher urinary excretion of mevalonic acid and higher activity of hydroxy-metil-coA reductase (HMG-coA) for the shunt of metabolic precursors towards the synthesis of pro-inflammatory isoprenoids [17]. This metabolic shunt could explain the efficacy of statines in reducing symptoms of HIDS, thanks to the anti-inflammatory effect of competiting inhibition of HMG-coA reductase [18].

Acute attacks can be triggered by physical stress or vaccination, can last 3 to 7 days and recur after 4 to 6 weeks. However a great variability can be observed as in many other periodic fever syndromes. Ninety per cent of HIDS patients experience first symptoms in the first year of life. Fever is usually associated with painful neck lymphadenopathy, abdominal pain, vomiting and diarrhea. About 50% of affected patients show aphtous oral or genital ulcers. Hepatosplenomegaly, maculopapular skin rash, nodules, urticarial or measle-like rash, arthralgias and non erosive arthritis of large joints can be present. Polyarticular arthritis is often remitting [2,3,11,19].

In the past years, high levels of IgD (> 100 UI/ml) during fever attacks was considered to be the main feature of HIDS, although their specificity is quite low: high levels of IgD can also be found in FMF or in TRAPS patients [20]. In some cases high levels of serum IgA and urinary mevalonic acid can be found during fever. Anyway urinary measure of mevalonic acid requires specialized laboratories, thus it is difficult to perform as a screening test. The decision to undergo a genetic test for HIDS in a child with periodic fever syndrome should be guided by clinical suspicion [2]. About 25% of patients with typical HIDS attacks and high serum IgD levels do not show any mutation of MVK gene [21].

During acute attacks the main treatment option are steroids (mainly prednisone in a single dose of 1 mg/day) [2]. Long term treatment in HIDS patients is quite controversial; in the literature there are only anecdotal cases of response to steroids, intravenous immunoglobulines, colchicine or cyclosporine A [14,22]. Thalidomide use has not proven to be efficacious while simvastatine use is quite encouraging in adult patients [14,23]. During the last years many studies have been reported on biologic drugs. Etanercept has been particularly studied for the pediatric age with controversial results [24-27]. In 2005 Bodar and coworkers showed variable response to etanercept in HIDS patients but in the same time anakinra has proven to be more effective in reducing the duration of symptoms [27]. Recent findings on biologics have shown that anakinra is the most effective in the control of fever attacks: new perspectives for HIDS treatment are now under judgement [28].

### TNF Receptor Associated Periodic Syndrome (TRAPS)

TRAPS is an auto- inflammatory multiorgan disease characterized by alternation of active and remission phases, which may be complicated with organ injury, in particular amyloidosis [29].

TRAPS, also known as Hybernian fever, was first described in 1982; it seems to be homogeneously distributed among different ethnic groups [11]; more than 200 cases are recorded in the international medical literature [3].

TRAPS has autosomal dominant inheritance; it is due to the mutations of *TNFRSF1A *gene, located on the short arm of chromosome 12. The gene encodes for the TNF membrane receptor (TNFR1); it consists of an extracellular domain that binds circulating TNF-α, a trans-membrane domain and an intracellular portion that transduce the signal inside the cell [30]. The wild-type TNFRSF1A protein interacts with circulating TNF-α and disconnects from the cell surface to neutralize the effect of the TNF-α; the mutant TNFRSF1A protein is unable to disconnect from the cell membrane, inducing continuous pro-inflammatory signal transmission by TNF-α [31].

TRAPS patients have episodes of recurrent fever associated with myalgia, migrant skin lesions, red, swollen, painful speckled localized to the trunk and extremities. Febrile attacks continue approximately two days but can last for weeks [2]. It is also common to find chest and abdominal pain, arthralgia of large joints, testicular pain, lymphadenopathy, headache, ocular involvement with periorbital edema and rarely aseptic conjunctivitis, uveitis or episcleritis [32].

TRAPS onset in pediatric age can be confused with juvenile idiopathic arthritis; it may also coexist with multiple sclerosis, panniculitis and vasculitis [33].

Acute episodes of TRAPS are characterized by a significant increase in acute phase reactants, neutrophilia and various degrees of hypochromic anemia [2]. The soluble TNF receptor levels are low both during attacks and during intercritical period [33]. Molecular studies showed about 80 variants of the *TNFRSF1A *gene, and about 64 of these have been identified in patients with a typical phenotype of TRAPS. The most common mutations are the R92Q and P46L, which show low penetrance, as demonstrated by their presence in asymptomatic relatives of TRAPS patients [34]. Furthermore, studies of genotype-phenotype correlation, even in the pediatric population [35], showed that mutations causing substitution of a cysteine residue are associated with a severe course of the disease and with increased possibility to develop renal amyloidosis.

TRAPS febrile attacks usually respond to steroids, but due to the possible long duration of episodes and the tendency to a chronic course, patients often become steroid-dependent [2]. The use of immunosuppressive drugs has proved ineffective in patients with TRAPS in reducing frequency and intensity of inflammatory episodes and in preventing the development of amyloidosis [33]. After the discovery of the underlying genetic defect, anti-TNF drugs, particularly etanercept, assumed a relevant role in the therapy of these disease [36]. The use of etanercept has proven to be effective in the control the symptoms and in some cases also in the improvement of nephrotic syndrome secondary to renal amyloidosis [32]. However, in some patients anti-TNF drugs, in particular infliximab, have been shown not fully able to control the inflammation [37]. On the contrary the use of anakinra seems to be an effective therapeutic strategy [38].

### Cryopyrinopaties (CAPS)

Cryopyrin-associated periodic syndromes encompass a spectrum of diseases characterized by recurrent inflammation and complications like renal amyloidosis, loss in sight, deafness and articular damage [31]. The term "cryopyrinopathies" clusters three syndromes all characterized by the mutation of *CIAS1 *gene:

• Familial Cold Autoinflammatory Syndrome (FCAS), first described in 1940;

• Muckle-Wells Syndrome (MWS), first described in 1962;

• Chronic Infantile Neurologic Cutaneous Articular Syndrome - Neonatal Onset Multisystem Inflammatory Syndrome (CINCA/NOMID), first described in 1981 by Prieur and Griscelli [2].

The CIAS1 gene is located on the long harm of chromosome 1 (1q44) and encodes for a protein called cryopyrin, which is involved in the formation of "inflammasome", a complex of protein that induced the activation of caspase 1 and the secretion of IL-1 [1,39,40].

FCAS is the mildest form. Its clinical features are fever spikes of short duration (typically less than 24hours), induced by exposure to cold and associated with profuse sweating, drowsiness, headache and nausea, conjunctivitis, and arthralgia [2]. FCAS attacks usually begin within the first few months of life [11].

MWS presents variable age of onset and is characterized by recurrent episodes of fever associated with urticaria: fever, usually less than 38°C, resolves spontaneously between 12 and 36 hours and is associated with the same symptoms of FCAS although it is never triggered by the exposure to low temperatures. MWS may be complicated by progressive hearing loss and amyloidosis even in childhood [2,31].

The CINCA/NOMID represents the most severe phenotype; it occurs mostly in the neonatal period or in early childhood, with an urticarial rash. One third of children have a typical "facies" including frontal prominence, saddle nose and facial hypoplasia; other skeletal features are abnormal bone and cartilage growth, especially in the distal extremities of hands, feet, knees and particularly of the patella (giant kneecap). Polyarticular chronic inflammation is often erosive and deforming [2]. This syndrome also includes the possible involvement of the central nervous system (aseptic chronic meningitis, progressive hearing loss, chronic headaches, mental retardation, epilepsy) and of eyes with anterior uveitis, papillitis and optic nerve atrophy [11,31,32].

The pathogenetic mechanisms of these diseases have allowed to identify anti IL-1 drugs as the most appropriate therapy. Anti IL-1 drugs, in particular anakinra (1 mg/Kg/day subcutaneously) are life saving drugs in patients with CAPS that usually respond to treatment within the first 24 hours [41]. Clinical studies have shown significant improvement in the clinical symptoms of CAPS including rash, headaches, fevers, joint pain and improvement in inflammatory markers with remission even in 60% of patients with CINCA/NOMID [42]. However, it may be difficult to achieve a complete resolution of neurologic involvement and high doses are often required [42,43]. Recent introduction of long acting IL-1inhibitors rilonacept and canakinumab also showed efficacy in patients with CAPS; both drugs were recently approved by Food and Drug Administration (FDA) for the treatment of CAPS [44,45].

### Blau syndrome

Blau syndrome was first described in 1985 as an autosomal-dominant autoinflammatory disease, familial or sporadic; it is caused by mutation in the NACHT domain of CARD15 (NOD2) (16q12.1-13) [2]. Disease onset is usually observed during the first years of life. It is characterized by non-caseating granulomatous lesions affecting joints (symmetric polyarthritis), skin and eyes, particularly the uveal tract. About 50% of patients with uveitis develop cataract, and one third may develop secondary glaucoma. Patients are treated with oral steroids and immunosuppressive drugs (methotrexate or cyclosporine A). Recently biologics are also used, especially anti-TNF (infliximab) and anti-IL1 drugs [2].

### PAPA syndrome

Pyogenic sterile arthritis, pyoderma gangrenosum and acne syndrome is caused by mutations in the *CD2BP1 *or *PSTPIP1 *(15q22-24) gene [2].

Arthritis onsets in early childhood and has been proved to be responsive to corticosteroids. It is destructive, primarily involving non-axial joints such as knees, elbows and ankles. Prominent cutaneous findings include cystic acne and ulcerative skin lesions, usually of lower extremities. Radiological examinations show articular lesions and signs of micrognathia and cervical ankylosis [46,47]. Cultures of the skin and joints of these patients are sterile with a neutrophilic infiltrate [46].

PAPA syndrome shows good response to oral glucocorticoids and biologics, particularly in steroid-resistant patient, with a dramatic improvement of cutaneous lesions [2].

### Majeed's syndrome: Chronic Recurrent Multifocal Osteomyelitis (CRMO)

Majeed's syndrome or CRMO is an auto-inflammatory osteopathy caused by mutations in *LPIN2 *gene (18p11.31) [2]. Symptoms begin in early childhood and are characterized by fever, bone lesions, pain, congenital dyserythropoietic anemia and dermatologic manifestations (psoriasis, palmoplantar pustulosis, acne).

The diagnosis of CRMO is based on the following critera [48]:

1. ≥2 typical bony lesions (osteolysis with surrounding sclerosis on conventional radiographs);

2. duration ≥6 months;

3. typical histologic features on bone biopsy;

4. age < 18 years at diagnosis.

Nonsteroidal antiinflammatory drugs and oral steroids are useful in the control of symptoms while splenectomy and blood transfusions have proven to be effective for controlling hematological manifestations [2].

### PFAPA syndrome

The acronym PFAPA is derived from the classical association of fever with pharyngitis, cervical lymphadenopathy and aphthous stomatitis. The disease was first described by Marshal in 1987; it usually develops before 5 years of age, with no evident correlations to ethnicity, geographical distribution or gender [5,49]. To date, no genetic defect has been associated to this syndrome.

PFAPA is characterized by fever spikes (body temperature of 38.5-41°C) that recur regularly every 2-6 weeks and last 3-6 days [2]. The affected child has a disease-free interval of about 3-5 weeks (albeit with great variability), and overall shows good general condition. The aphthous lesions observed in PFAPA are generally one to five, small and not keratinized. They are localized in the labial gingiva and are rapidly self-remitting [2,49]. Cervical lymphnode are enlarged and tender. In some cases pharyngitis is associated with follicular exudative tonsillitis with negative cultural examination for group A streptococcus and in the absence of signs of upper respiratory infections.

The child with PFAPA can also present headache, general malaise, arthritis, gastrointestinal disorders (particularly nausea and vomiting), splenomegaly in contrast to a normal somatic growth and normal development [49].

There are not specific diagnostic tests for PFAPA, so the diagnosis is based on clinical evaluation and exclusion of other causes. Laboratory testings may reveal mild leukocytosis and elevated erythrocytes sedimentation rate only during febrile episodes. Besides, mild elevations in IgA, IgD and IgM are described in some patients [50]. In the absence of clinical clues suggesting for an hereditary syndrome, there is no need of genetic testing. Only in patients with high diagnostic score the option of genetic analysis should be considered [4].

Treatment of PFAPA is empirical, as the underlying cause is unknown. The most effective treatment reported to date is prednisone or prednisolone at the dose of 1-2 mg/Kg/day at the onset of fever [50]. Cimetidine has been associated with resolution of the febrile episodes [51,52] although the mechanism of action of this drug in PFAPA has not been determined. Probably the action of cimetidine is linked to the inhibition of T suppressor cells by blocking histamine type 2 receptors [50]. Some patients should be considered for tonsillectomy and adenoidectomy if they do not respond to medical management [53,54].

However, the lack of controlled trials should lead to caution in interpreting the apparently successful treatment of PFAPA by cimetidine or tonsillectomy.

## Conclusions

In children with a suspicion of a periodic fever syndrome, an accurate clinical history and a physical examination remain the first diagnostic tools. In many cases the diagnosis may not be immediate; long term follow up is seldom of primary importance to clarify the disease course and to guide in the diagnostic process. Differential diagnosis includes infectious diseases, malignancies and organ-specific inflammatory syndromes. Only in the absence of other conditions, an autoinflammatory syndrome should be considered. The genetic testing to date has not replaced clinical diagnosis; it should be performed only in highly suggestive cases as discussed before. Furthermore only 50% of affected patients have positive genetic tests, suggesting that many genetic loci are still unknown. In the future genome wide association studies may help in the recognition of candidate genes. Future understandings may also change therapeutic perspectives, allowing targeted drugs mostly for those syndromes that today are only empirically treated.

## Abbreviations

FMF: Familial Mediterranean Fever; HIDS: Hyper-IgD Syndrome; TRAPS: Tumor necrosis factor Receprtor associated Autoinflammatory Periodic Syndrome; CAPS: Cryopirin Associated Periodic Syndromes; PAPA: Pyogenic sterile Arthritis Pyoderma gangrenosum and Acne syndrome; PFAPA: Periodic Fever, Aphtous stomatitis, Pharyngitis and Adenitis; CRMO: Chronic Recurrent Multifocal Osteomyelitis; NSAIDS: Non Steroidal Anti Infammatory Drugs; IL-1: Interleukin 1; MEFV: MEditerranean FeVer; CRP: C-Reactive Protein; SAA: Serum Amyloid A; TNF-α: Tumor Necrosis Factor alpha; MVK: mevalonate kinase; HMG-COA: Hydroxy-Methil-Glutharil Coenzyme A reductase; FCAS: Familial Cold Autoinflammatory Syndrome; MWS: Muckle Wells Syndrome; CINCA/NOMID: Chronic Infantile Neurologic Cutaneous Articular syndrome/Neonatal Onset Multisystem Inflammatory Disease.

## Competing interests

The authors declare that they have no competing interests.

## Authors' contributions

All authors equally contributed in the acquisition of data from the International literature, in drafting the manuscript and revising the final text. All authors read and approved the final manuscript.
